# Ubiquitin is a carbon dioxide–binding protein

**DOI:** 10.1126/sciadv.abi5507

**Published:** 2021-09-24

**Authors:** Victoria L. Linthwaite, Wes Pawloski, Hamish B. Pegg, Philip D. Townsend, Michael J. Thomas, Victor K. H. So, Adrian P. Brown, David R. W. Hodgson, George H. Lorimer, David Fushman, Martin J. Cann

**Affiliations:** 1 Department of Biosciences, Durham University, Durham DH1 3LE, UK.; 2 Department of Chemistry and Biochemistry, Center for Biomolecular Structure and Organization, University of Maryland, College Park, MD 20742, USA.; 3 Department of Chemistry, Durham University, Durham DH1 3LE, UK.; 4 Biophysical Sciences Institute, Durham University, Durham DH1 3LE, UK.; 5 Biophysics Program, Institute for Physical Science and Technology, University of Maryland, College Park, MD 20742, USA.

## Abstract

The identification of CO_2_-binding proteins is crucial to understanding CO_2_-regulated molecular processes. CO_2_ can form a reversible posttranslational modification through carbamylation of neutral N-terminal α-amino or lysine ε-amino groups. We have previously developed triethyloxonium (TEO) ion as a chemical proteomics tool for covalent trapping of carbamates, and here, we deploy TEO to identify ubiquitin as a mammalian CO_2_-binding protein. We use ^13^C-NMR spectroscopy to demonstrate that CO_2_ forms carbamates on the ubiquitin N terminus and ε-amino groups of lysines 6, 33, 48, and 63. We demonstrate that biologically relevant *p*CO_2_ levels reduce ubiquitin conjugation at lysine-48 and down-regulate ubiquitin-dependent NF-κB pathway activation. Our results show that ubiquitin is a CO_2_-binding protein and demonstrates carbamylation as a viable mechanism by which mammalian cells can respond to fluctuating *p*CO_2_.

## Teaser

Altered ubiquitin conjugation is a mechanism by which mammalian cells can respond to fluctuating *p*CO_2_.

## INTRODUCTION

Carbon dioxide is an absolute requirement for life. However, we know relatively little of the mechanisms that underpin direct interactions of CO_2_ with the cell, despite the importance of the gas to biology. The only identified signaling molecules that respond directly to inorganic carbon [excluding a potential signaling role for carbonic anhydrases (*1*)] are the class III nucleotidyl cyclases of animals, fungi, and prokaryotes (*2*–*5*); a subset of connexins (typified by Cx26) in mammals (*6*); and receptor protein tyrosine phosphatase γ of mammals (*7*).

One hypothesis for how CO_2_ regulates signaling is that it mediates a protein posttranslational modification (PTM); CO_2_ might regulate the activity of multiple proteins and signaling pathways. There is direct evidence that CO_2_ and protein can interact through carbamylation of neutral lysine ε-amino- and N-terminal α-amino groups and affect the activities of RuBisCO (*8*) and hemoglobin (Hb) (*9*), respectively. Several proteins carry a stable carbamate required for catalysis, e.g., urease, alanine racemase, transcarboxylase 5S, class D β-lactamase, and phosphotriesterase (*10*). Reversible carbamylation of neutral protein amino groups could yield responses to fluctuating *p*CO_2_ (partial pressure of CO_2_) that might, therefore, constitute a widespread mechanism for protein regulation (*10*, *11*). The hypothesis centers on the dissociation of cationic ammonium groups to neutral amines within structurally privileged environments within CO_2_-responsive proteins. Carbamate formation, mediated by nucleophilic attack of the neutral amines on CO_2_, leads to the formation of anionic groups, with the possibility for profound biological consequences as evidenced in Hb and RuBisCO. Our previous work developed the use of the triethyloxonium (TEO) ion as a tool to covalently trap the carbamate PTM on the protein (*10*). TEO is a water-soluble reagent that traps carbamates by selective alkylation. TEO has a *t*_1/2_ of ~6 min at pH 7.4 under aqueous conditions. This *t*_1/2_ permits its use as a trapping agent with mixing and pH control on a convenient laboratory time scale and we have used TEO as a tool to identify new CO_2_-binding proteins. Here, we have deployed TEO to identify mammalian CO_2_-binding proteins and identified ubiquitin (Ub) as a CO_2_-binding protein.

Ub is a highly conserved 8.5-kDa protein found in all eukaryotic cells, regulating protein activity and degradation through conjugation to target proteins. The identification of Ub as a CO_2_-binding protein can explain how CO_2_ has these diverse effects in mammalian cells. The ubiquitination PTM involves Ub protein covalent conjugation to a lysine side chain on target proteins. Conjugation of Ub into poly-Ub chains can potentially occur at every Ub lysine side chain and the N-terminal α amino group producing eight functionally distinct chain formations. These varying linkages underpin different physiological processes (*12*). The well-characterized poly-Ub chains linked via lysine-48 result in protein targeting to the proteasome for degradation (*12*), while lysine-63–linked chains regulate proteasome-independent reactions, including endocytosis (*13*). Poly-Ub conjugates at other lysine residues can affect the mammalian cell cycle (Lys^11^) and 5′ AMP-activated protein kinase (AMPK)–related kinases to regulate enzymatic activity (Lys^29^ and Lys^33^) (*14*).

## RESULTS

### TEO ion traps CO_2_ on Ub

A previous TEO-based chemical proteomics screen of *Arabidopsis thaliana* whole protein lysate in the presence of 20 mM NaHCO_3_ identified seven CO_2_-binding proteins (*10*). A further screen identified open reading frame At3g52590 as a potential CO_2_-binding protein. At3g52590 encodes the UBQ1 Ub extension fusion protein (*15*). Therefore, we hypothesized that if CO_2_-binding sites within proteins are specific and conserved, Ub will represent a suitable candidate mammalian receptor for CO_2_.

**Fig. 1.**
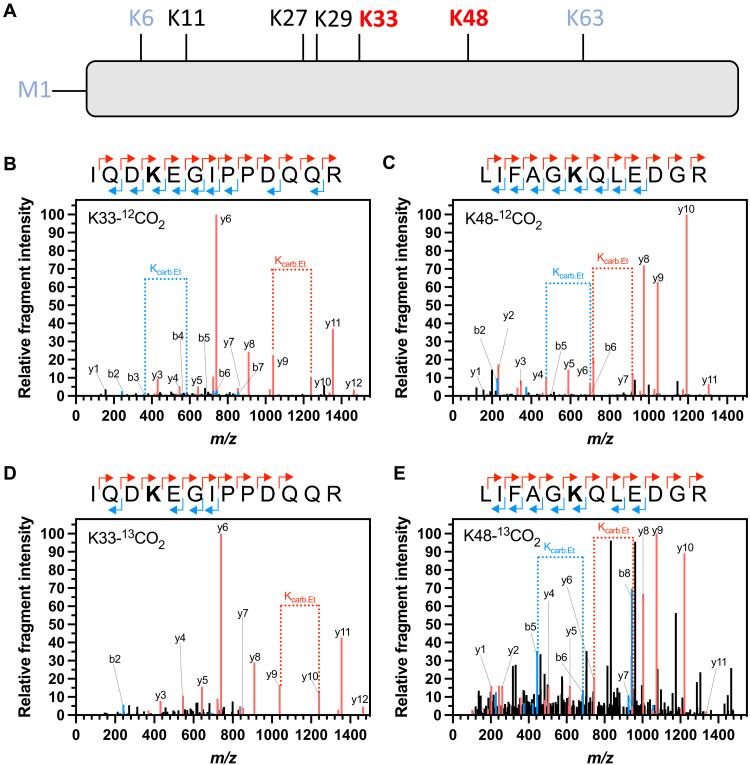
CO_2_ is covalently trapped on Ub. (**A**) Cartoon of Ub, demonstrating the seven conserved lysine ubiquitination sites and the N-terminal M1 site. Those identified as carbamylated through both MS-MS and ^13^C-NMR are shown in bold red type. The sites identified as carbamylated through ^13^C-NMR are shown in blue type. (**B** to **E**) Identification of exchangeable CO_2_-binding sites on Ub by MS-MS. Plots of relative fragment intensity versus *m/z* for fragmentation data from MS-MS identifying ethyl-trapped carbamate on Ub K33 (B and D) and K48 (C and E) in the presence of ^12^CO_2_ (B and C) or ^13^CO_2_ (D and E). Peptide sequences indicate the identification of predominant +1y (red) +1b (blue) ions by MS-MS shown in the plot. The modified residue is displayed in bold. K_carb.Et_ indicates the molecular weight difference between ions diagnostic of the modified Lys.

A key feature of Ub is its seven conserved lysine residues (K6, K11, K27, K29, K33, K48, and K63) and its N-terminal α-amino group, which can serve as ubiquitination sites in the formation of poly-Ub chains (Fig. 1A). Ubiquitination influences a spectrum of cellular processes by regulating protein function, fate, and subcellular localization (*16*). We expressed the human Ub protein as a recombinant protein in *Escherichia coli*. Ub was equilibrated with 25 mM CO_2_/HCO_3_ at pH 7.4, and TEO was added. Trypsin was used to digest the trapping reaction mixture, and liquid chromatography–tandem mass spectrometry (LC-MS-MS) was used to analyze samples, followed by data analysis using Peaks (Bioinformatics Solutions Inc.). The data were interrogated for modifications on the N terminus and lysine with masses of 72.0211 Da (trapped carbamate) and 28.0313 Da (*O*-ethylation on glutamate and aspartate side chains). Two lysine carbamylation sites were identified (MS-MS peptide amino acids 30 to 42 IQDKEGIPPDQQR, proposed carbamylation on K33; MS-MS peptide amino acids 43 to 54 LIFAGKQLEDGR, proposed carbamylation on K48) (Fig. 1, B and C). Within the datasets presented here, the carbamates were observed on both peptides on internal lysine residues that both exhibited a so-called missed cleavage. The missed cleavage is because carbamylation removes the cationic charge on the lysine essential for cleavage site recognition by trypsin. This supports the identification of carbamates on both Ub K33 and K48 as a missed cleavage is an otherwise rare event. The datasets also include peptides cleaved at K33 and K48, and these peptides do not carry a trapped carbamate. We performed the CO_2_-trapping experiments on Ub with 25 mM ^13^CO_2_/H^13^CO_3_ to corroborate the carbamate PTMs by interrogating the MS-MS data for a 73.0211-Da modification. The expected +1-Da mass/charge ratio (*m/z*) increase was observed for the carbamylation sites at both K33 (Fig. 1D) and K48 (Fig. 1E). MS-MS peptides encompassing the N-terminal α-amino group, K6, K11, K27, K29, and K63, were observed, but no potential carbamylation sites were identified by this method.

### Observation of carbamate formation on Ub by ^13^C-NMR

**Fig. 2.**
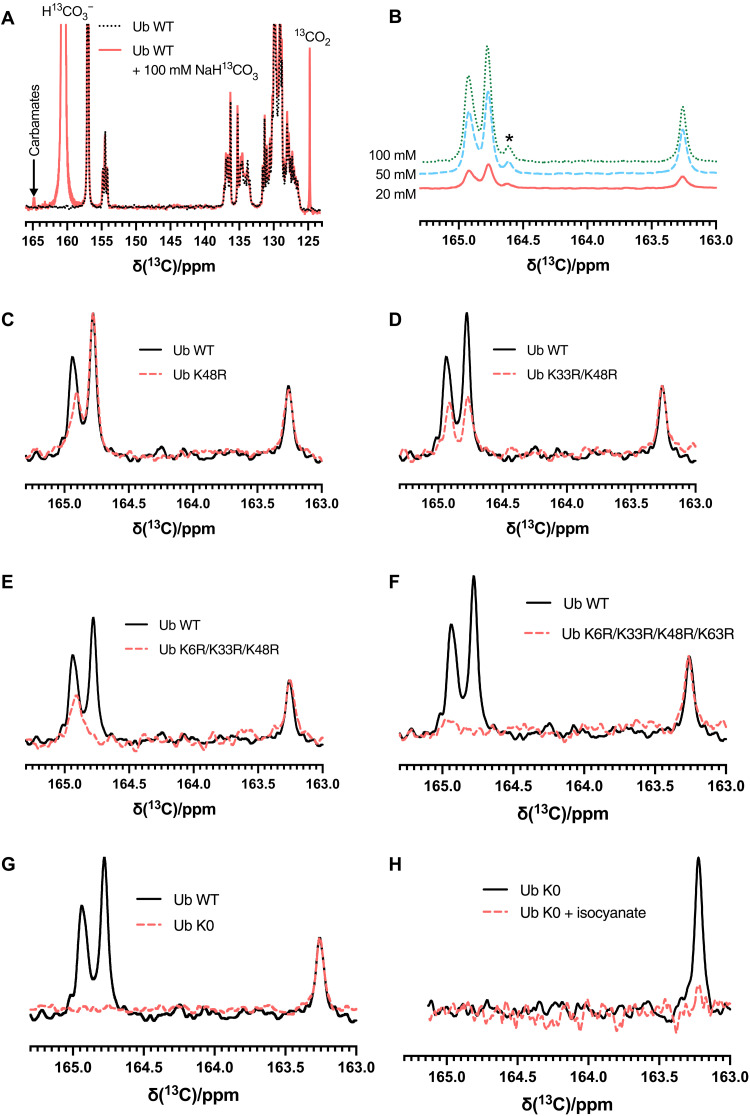
CO_2_ forms carbamates on Ub. (**A**) Ub carbamates are observed with NaH^13^CO_3_. Two 1D ^13^C-NMR spectra of ^15^N/^13^C-labeled Ub WT and the same sample exchanged into an identical buffer with 100 mM NaH^13^CO_3_ are overlaid. The background ^13^CO_2_ and H^13^CO_3_^−^ are observed along with carbamates. (**B**) The carbamate signal intensity is affected by the NaH^13^CO_3_ concentration. The carbamate region from 1D ^13^C-NMR spectra is shown for buffers prepared with 5 mM Ub WT varying NaH^13^CO_3_ concentration, and the intensities were standardized to the Arg C_ζ_ resonances. The carbamate signals were identified by consecutive Lys to Arg mutations to make Ub mutant variants K48R (**C**), K48R/K33R (**D**), K48R/K33R/K6R (**E**), K48R/K33R/K6R/K63R (**F**), and K0 (all seven Lys residues mutated to Arg). (**G**) K0 Ub was mixed with one equivalent of sodium cyanate to carbamoylate the N-terminal amine and compared to unmodified K0 Ub. A low-intensity ^13^C carbamate signal [marked with * in (B)] is observed at 5 mM Ub, but not 1 mM Ub, from the same batch (fig. S1) or other Ub mutants (C to G); the signal could be attributed to a minor population of carbamates on other Ub lysines or on a noncovalent Ub dimer at this concentration [dissociation constant (*K*_d_) ~ 4.9 mM] (*59*).

We used ^13^C-NMR (nuclear magnetic resonance) as an orthologous method to confirm Ub CO_2_-binding sites at K33 and K48, to investigate other carbamate formation sites not identified by MS-MS, and as a direct demonstration of the carbamate PTM on native protein. We initially mixed 1 mM ^13^C/^15^N-labeled Ub with 100 mM NaH^13^CO_3_ and observed three peaks in 1D-^13^C NMR spectra, which were not present in spectra for either Ub or NaH^13^CO_3_ alone (Fig. 2A). These new signals’ chemical shifts—163.25, 164.77, and 164.96 ppm—are consistent with the empirical range for carbamate PTMs (*17*). Unlabeled Ub (1 or 5 mM) was subsequently exchanged into buffers containing 20, 50, or 100 mM NaH^13^CO_3_, and we observed that the intensities of these carbamate signals increased with increasing [NaH^13^CO_3_] or Ub concentration (Fig. 2B and fig. S1), supporting the hypothesis that they are the product of reversible carbamate formation on Ub. The ratios of the peak intensities were unaltered at 20, 50, or 100 mM NaH^13^CO_3_. We therefore used 100 mM NaH^13^CO_3_ to identify the carbamate-forming residues. Experiments performed with the double mutant Ub K48R/K6R yielded partial reductions in the two signal intensities at 164.77 and 164.96 ppm (fig. S2A). In a duplicate experiment with uniformly ^15^N-labeled Ub K48R/K6R, these remaining signals were split into doublets; the observed splittings of ~18.5 Hz were consistent with a one-bond ^15^N-^13^C coupling (fig. S2B), indicating that the ^13^C atoms responsible for these signals are directly bonded to ^15^N atoms, and confirm that these are carbamate signals. To unambiguously identify the carbamate signals, we performed consecutive lysine to arginine Ub mutations starting with K48R (Fig. 2C) and adding K33R (Fig. 2D), K6R (Fig. 2E), and K63R (Fig. 2F), which revealed that the signals at 164.77 and 164.96 ppm were constituted of overlapping carbamate signals from K6/K33 and K48/K63, respectively. The ^13^C-NMR spectrum of the quadruple mutant (K6R/K33R/K48R/K63R) matched the spectrum of a lysine-free Ub (K0) (Fig. 2G). This observation demonstrates that carbamylation was not evident at K11, K27, or K29. Removal of all Ub lysines in Ub K0 did not affect the ^13^C-NMR carbamate resonance at 163.25 ppm. The only potential nucleophilic nitrogen atoms remaining in Ub K0 are the N-terminal amino group and, possibly, the imidazole of H68. N-acetyl-l-histidine cannot form adducts with CO_2_ (*18*) but is identified in the binding pockets of proteins that can interact with CO_2_ (*19*). An H68A mutation was introduced into Ub to examine whether H68 (the single histidine residue in Ub) was responsible for this remaining signal. This mutation altered the population of carbamylated K6, likely reflecting an altered p*K*_aH_ of K6 due to the proximity of its ε-amino group to the imidazole of H68, but did not affect the resonance at 163.25 ppm (fig. S3). We reacted Ub K0 with one equivalent of sodium cyanate, which ablated the carbamate resonance at 163.25 ppm, identifying it as originating from carbamylation of the N-terminal amine (residue M1; Fig. 2H).

The p*K*_aH_ value for the lysine ε-amino group is substantially above physiological pH. As carbamylation depends on the dissociation of the ε-amino moiety to a neutral group, those lysines with lower ε-amino p*K*_aH_ values are potentially more likely to form carbamates. We therefore determined the p*K*_aH_ for lysine ε-amino groups and the N-terminal amine in Ub. Lysine-specific N_ζ_ or C_ε_ chemical shift data were obtained over the pH range from 8.6 to 12.3 and fit to a Henderson-Hasselbalch model to extract the p*K*_aH_ for each ε-amino group (and the α-amino group of M1) (fig. S4). The data demonstrate an ordering of p*K*_aH_ of K6 < K48 < K33 < K63 < K27 < K11 < K29 (Table 1), in agreement with a recent report (*20*). The observation of carbamylation on K6, K33, K48, and K63 using ^13^C NMR, therefore, matches the ordering of p*K*_aH_ for each ε-amino group. In conclusion, human Ub has been identified, through MS-MS and ^13^C-NMR spectroscopy, to be capable of binding CO_2_ through carbamate PTM formation.

**Table 1.** p*K*_aH_ values for Ub lysine ε-amino groups and -amine of N-terminal M1. α The reported errors in p*K*_aH_ values reflect errors in determining the ^15^N or ^13^C chemical shifts alone and do not account for errors in pH measurements at elevated pH (see Supplementary Materials and Methods).

**Residue**	**p*K*_aH_**
M1	9.19 ± 0.05
K6	10.47 ± 0.02
K48	10.53 ± 0.02
K33	10.88 ± 0.03
K63	11.03 ± 0.02
K27	11.2 ± 0.1
K11	11.47 ± 0.03
K29	11.68 ± 0.03

### Carbamate formation down-regulates Ub conjugation at K48 in vitro

We hypothesized that Ub carbamylation would affect poly-Ub formation. Ubiquitination occurs through the sequential activity of a Ub-activating enzyme (E1), a Ub-conjugating enzyme (E2), and a Ub ligase (E3) (*21*). E1 forms a thioester between a catalytic cysteine and glycine-76 at the Ub C terminus. E1 transfers Ub via G76 to a catalytic cysteine on E2 and forms an E2-Ub thioester complex. E3s bind this complex and substrate and enable formation of an isopeptide bond between the Ub C-terminal carboxyl group and the ε-amino group of a substrate lysine or an N-terminal amino group. Successive reaction rounds can produce poly-Ub chains linked via the seven Ub lysine residues or the N terminus of M1. We hypothesized that carbamate formation on Ub would alter the charge/binding capacity of the modified lysine ε-amino group and therefore down-regulate poly-Ub chain formation by blocking the transfer of free Ub onto a target Ub molecule. Ub conjugation at K48 is well characterized in vitro and in vivo, particularly concerning proteasomal function (*22*). We identified K48 as a site for carbamylation by TEO-trapping (Fig. 1, C and E) and ^13^C-NMR spectroscopy (Fig. 2C). Therefore, we selected Ub conjugation at K48 to investigate the biochemical relevance of Ub carbamylation. A carbamate was identified on Ub K63 by ^13^C-NMR spectroscopy (Fig. 2F), but not TEO trapping. Therefore, we selected Ub conjugation at K63 as an additional site for analysis. Conjugation assays at both Ub K48 and Ub K63 used the mE1 protein as a Ub-activating enzyme (*23*). Ub conjugation at specific lysine side chains can be investigated using E2 and E3 enzymes specific for conjugation at that site. Conjugation at Ub K48 used the E2-25K protein, which functions as both an E2 and an E3 enzyme, while conjugation at Ub K63 used the UEV1-Ubc13 heterodimer. These assays were performed over a concentration range that incorporated physiologically relevant CO_2_ concentrations (a reference range of 1.8 to 2.3 mM dissolved CO_2_ corresponding to a *p*CO_2_ of 4.6 to 6.0 kPa) as well as pathophysiological hypocapnic CO_2_ (<1.8 mM dissolved CO_2_) and up to severe pathophysiological hypercapnic CO_2_ (3.0 mM dissolved CO_2_) (*24*). pH was monitored before, during, and at the end of each assay and was within ±0.1 pH units. Any observations are therefore independent of pH.

**Fig. 3.**
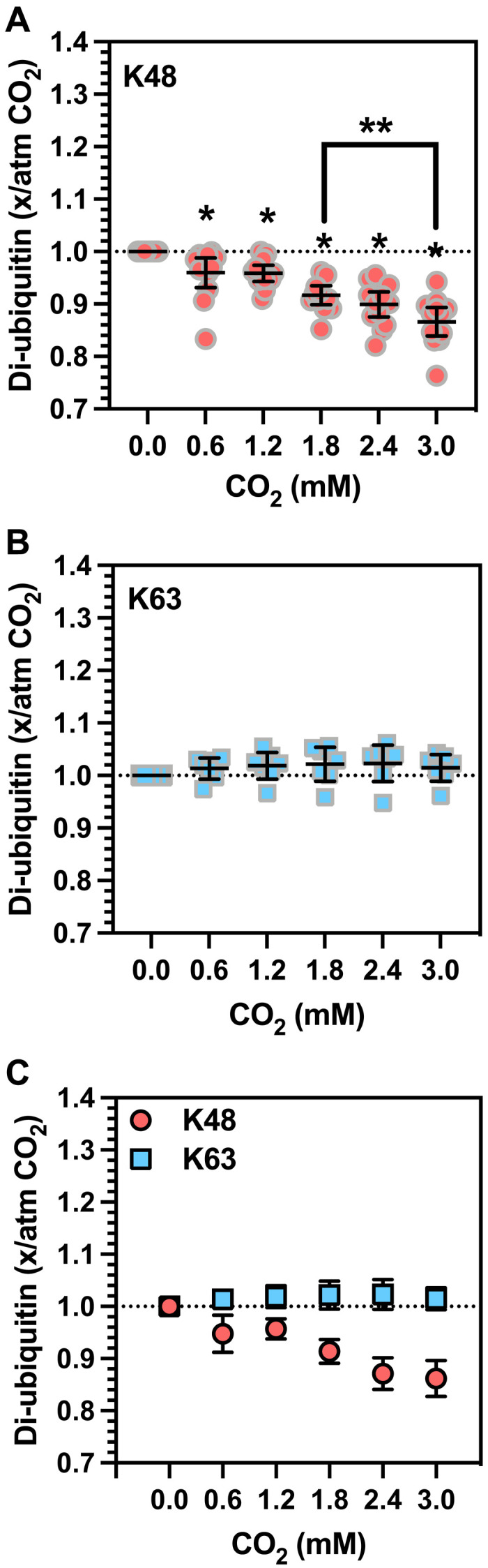
CO_2_ modifies Ub conjugation at K48 in vitro. A plot of the ratio of di-Ub formed in the presence of added CO_2_ compared to atmospheric CO_2_ against the concentration of added CO_2_. Values are normalized to 1 using the value at 1.8 mM CO_2_. 0 corresponds to atmospheric CO_2_. (**A**) Ub conjugation at K48 [mean ± 95% confidence interval (CI); *P < 0.01, one-sample *t* test, theoretical mean = 1.000, *t* > 3.122, df = 12]. (**B**) Ub conjugation at K63 (mean ± 95% CI). (**C**) Comparison of data for K48 and K63 conjugation (mean ± 95% CI).

We observed an approximate 12% decrease in di-Ub formation at K48 over 0.0 to 3.0 mM CO_2_ (Fig. 3, A and C), consistent with an inhibition of E2-25K activity due to the carbamate on Ub K48. An increase in CO_2_ from 1.8 to 3.0 mM CO_2_ (corresponding to in vivo hypercapnia) revealed decreased di-Ub formation at K48. Reductions in CO_2_ below 1.8 mM (corresponding to in vitro hypocapnia) showed increased di-Ub formation at K48. We observed no change in di-Ub formation at K63 over 0.0 to 3.0 mM CO_2_ (Fig. 3, B and C). Analysis of the data for di-Ub formation at K48 by one-way analysis of variance (ANOVA) demonstrated a significant decrease in di-Ub formation between 1.8 and 3.0 mM CO_2_. Di-Ub formed represented ~30% of the input Ub, suggesting that the observations are robust and not reflective of a minor reaction. Thus, experiments in vitro demonstrate that pathophysiologically relevant changes in CO_2_ significantly alter Ub conjugation at K48.

It is formally possible that the influence of altered CO_2_ on Ub conjugation at K48 is due to CO_2_ binding to an alternative assay component to Ub. The Ub-activating enzyme, mE1, is common for conjugation at both K48 and K63 and is therefore unlikely to be a CO_2_ target due to no observed influence of CO_2_ in the K63 conjugation assay. We used TEO-based trapping in an attempt to identify a potential CO_2_-binding site on E2-25K to investigate the possibility that the E2/E3 enzyme for K48 conjugation is a CO_2_ target. TEO trapping and subsequent MS-MS failed to identify any E2-25K peptides with trapped carbamates (174 peptides with 94% coverage of the E2-25K protein).

Note that CO_2_ did not influence K63 conjugation under the assay’s conditions. The carbamate on K48 may be more stable than that on K63, but this awaits further investigation. Therefore, while carbamates on both K48 and K63 are detectable by ^13^C-NMR, we propose that only the carbamate at K48 has a sufficient residence time to influence Ub conjugation over the time scale of the in vitro assay.

### Carbamate formation down-regulates Ub conjugation at Ub K48 in cellulo

We hypothesized that exposure of cells to elevated CO_2_ would affect Ub-dependent processes in the cell. Ubiquitination of proteins regulates nuclear factor κB (NF-κB) signaling (*17*). Two NF-κB activation pathways have been described: the canonical (classical) and noncanonical (alternative) pathways. Various ligands, including tumor necrosis factor–α (TNF-α), associated with local inflammatory and immune responses, induce the activation of the canonical NF-κB pathway. NF-κB is maintained in the nonactivated state in the cytoplasm through binding to the inhibitor of NF-κB (IκB) proteins. Phosphorylation of IκB proteins results in ubiquitination with K48-linked poly-Ub and subsequent degradation of IκB by the proteasome. NF-κB is subsequently transported to the nucleus, where it activates a transcriptional response.

**Fig. 4.**
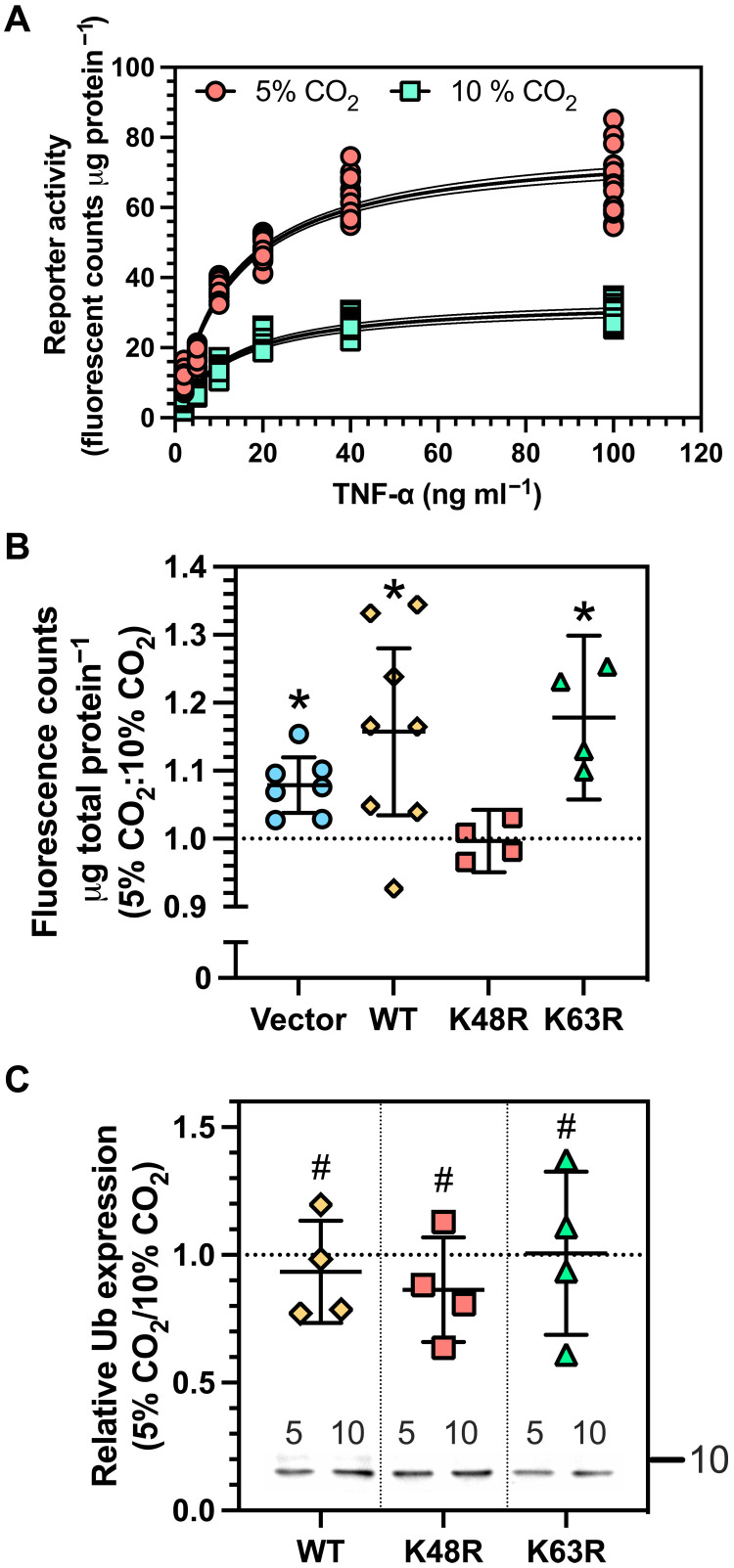
CO_2_ modifies signaling dependent on K48-linked poly-Ub in cellulo. (**A**) Plot of the fluorescence reporter activity for untransfected NF-κB/293/GFP-Luc cells against TNF-α at 5 and 10% (v/v) CO_2_. The dotted line indicates the 95% confidence interval. (**B**) Plot of the ratio of fluorescence reporter activity for transfected NF-κB/293/GFP-Luc cells at 5 and 10% (v/v) CO_2_ treated with TNF-α (30 ng ml^−1^). Cells were transfected with vector only or with a plasmid expressing WT Ub (WT), mutant K48R Ub (K48R), or mutant K63R Ub (K63R) (mean ± 95% CI; *P < 0.02, one-sample *t* test, theoretical mean = 1.000, *t* > 3.027, df = 3 to 7). (**C**) Plot of the ratio of Ub expression at 5% (v/v) versus 10% (v/v) CO_2_ for three independent samples from (B) expressing WT, K48R, or K63R Ub (mean ± 95% CI; ^#^*P* > 0.05, one-sample *t* test, theoretical mean = 1.000, *t* < 1.896, df = 2). The immunoblot below shows mono-Ub protein detected with an α-HA-tag antibody at the indicated CO_2_ level (% v/v).

Elevated CO_2_ suppresses NF-κB–mediated transcription (*25*–*28*). This suppression is proposed to have therapeutic potential (*29*) but remains controversial (*30*). Regardless of the controversy, the mechanism(s) by which CO_2_ influences NF-κB–mediated transcription is unknown. We investigated whether Ub was able to determine responses of NF-κB–mediated transcription to CO_2_. Experiments used human embryonic kidney (HEK) 293 cells (NF-κB/293/GFP-Luc) transduced with HIV-based pseudoviral particles packaged with a lentivector that coexpressed destabilized copGFP [but whose stability is not altered by pH (*31*)] driven by the minimal cytomegalovirus promoter (mCMV) in conjunction with four copies of the NF-κB consensus transcriptional response element upstream of mCMV. We exposed NF-κB/293/GFP-Luc cells to increasing concentrations of TNF-α under culture media equilibrated to normocapnic [5% (v/v) CO_2_] or hypercapnic [10% (v/v) CO_2_] conditions (endpoint pH 7.5) (Fig. 4A). Physiological hypercapnia occurs above 45 mmHg *p*CO_2_. We selected cell culture conditions of 10% (v/v) CO_2_ as representative of CO_2_ levels encountered in disease (*32*). Extracellular pH was monitored before, during, and after assays and was constant across all conditions. Cells were permitted to undergo intracellular pH (pHi) homeostasis using our previously established methodology to ensure that changes in pHi did not influence the results (*33*). Resting pHi for HEK 293 cells (~7.4) is consistent with our in vitro assay conditions (*34*). NF-κB–dependent green fluorescent protein (GFP) reporter activity was suppressed at 10% (v/v) compared to 5% (v/v) CO_2_ as hypothesized.

We transfected NF-κB/293/GFP-Luc cells with plasmids encoding wild-type (WT) Ub, a mutant K48R Ub, a mutant K63R Ub, or an empty vector. We hypothesized that overexpression of K48R Ub would alter the relative response of the NF-κB pathway to elevated CO_2_. In contrast, for the cases of WT Ub and Ub K63R, we expected the NF-κB response to be insensitive to CO_2_ in vitro (Fig. 3, B and C). We observed a ratio of fluorescence reporter activity at 5% (v/v) compared to 10% (v/v) >1 in vector-transfected cells, consistent with a reduction in NF-κB–dependent transcription (Fig. 4B). A similar observation was made in cells transfected with WT or a K63R Ub. However, we observed a ratio of fluorescence reporter activity at 5% (v/v) compared to 10% (v/v) not significantly different from 1.0 in K48R-transfected cells, consistent with no change in NF-κB–dependent transcription. This finding suggests Ub K48 to be the target for CO_2_ in the NF-κB–dependent transcriptional response to hypercapnia. We speculate that K48R Ub might be introduced into endogenous Ub chains at a rate sufficient to permit eventual fluorescence reporter activation and ablate the impact of CO_2_ on poly-Ub formation. Analysis of the ratio of production of the transfected Ub protein at 5% (v/v) versus 10% (v/v) CO_2_ demonstrated no significant difference between WT Ub, K48R Ub, and K63R Ub; thus, differences in protein production do not explain these results (Fig. 4C). A faint band was visible above the predominant signal in the WT Ub–transfected sample. The identity of the protein in this band is not known, but its density does not alter the experimental findings. Data were reported as ratios of fluorescence reporter activity at 5% (v/v) compared to 10% (v/v) CO_2_, as the variation in raw values for fluorescence reporter activity was greater than the change in ratio. All values were normalized to total loaded protein. This variation was likely due to variation in Ub plasmid transfection efficiency and Ub protein production. We cannot, therefore, rule out whether the influence of CO_2_ on reporter activity occurs specifically at 5% (v/v) versus 10% (v/v) CO_2_ (or both).

**Fig. 5.**
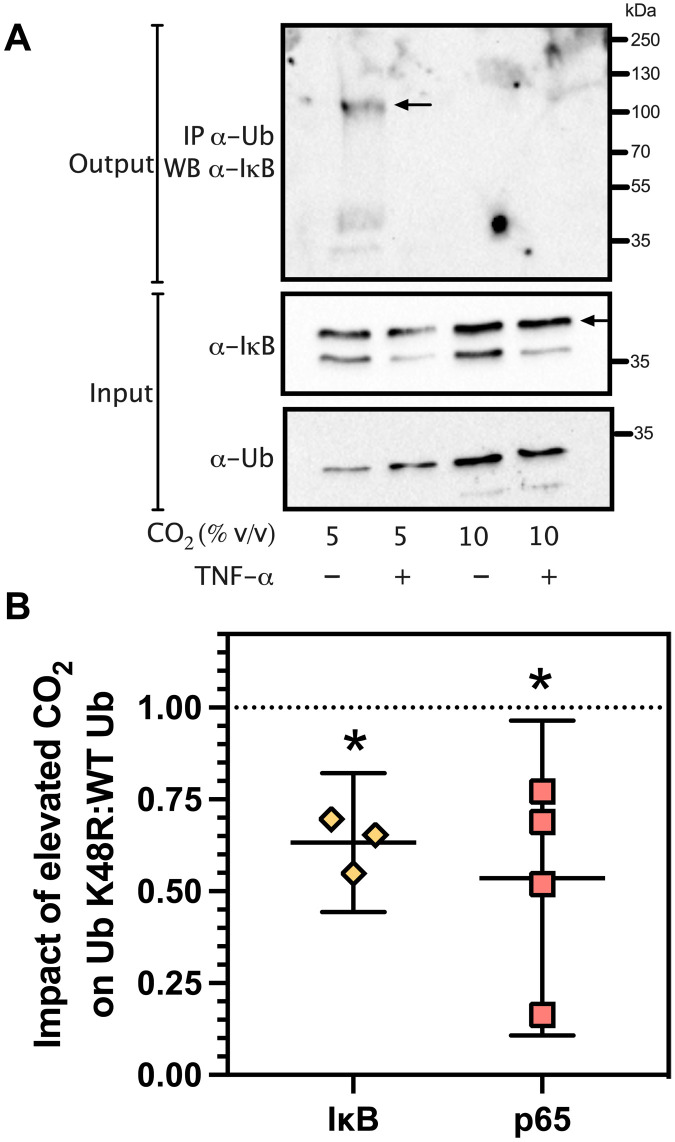
K48-linked poly-Ub alters sensitivity to CO_2_ in cellulo. (**A**) Interaction of IκB with Ub. Coimmunoprecipitation of endogenous IκB and Ub from HEK 293 cells. The labels on the figure are as follows: Input: immunoblot of endogenous IκB and Ub; IP α-Ub WB α-IκB: immunoblot performed using an α-IκB antibody after immunoprecipitation with an α-Ub antibody; CO_2_ (% v/v): gas conditions under which the experiment was performed; TNF-α: presence or absence of TNF-α (30 ng ml^−1^); arrow in α-IκB, Input: IκB protein; arrow in top panel: IκB-Ub conjugate; kDa: molecular weight markers. See also fig. S4. (**B**) Plot of the impact of elevated CO_2_ in HEK 293 cells transfected with Ub K48R compared to Ub WT on the ratio of the response for IκB degradation and p65 nuclear localization (mean ± 95% CI; *P < 0.05, one-sample *t* test, theoretical mean = 1.000, *t* > 3.447, df = 2 to 3).

We investigated whether Ub conjugation on IκB was sensitive to elevated CO_2_. IκB is conjugated with Ub under basal cell conditions that form a high–molecular weight complex, and activation of the NF-κB pathway can enhance this conjugation (*35*). We treated HEK 293 cells with or without TNF-α at 5% (v/v) versus 10% (v/v) CO_2_. TNF-α treatment was optimized such that bulk IκB was not degraded and thus able to be analyzed for Ub conjugation. Western blot analysis demonstrated approximately equivalent amounts of endogenous Ub and IκB under the varying conditions (Fig. 5A, α-IκB and α-Ub Input, and fig. S5). A faint band was visible below the predominant signal in the 10% (v/v) CO_2_ input sample analyzed with an α-Ub antibody. The identity of the protein in this band is not known, but its density does not alter the experimental findings. We analyzed IκB by Western blot after immunoprecipitation with α-Ub antibody to identify an IκB-Ub conjugate. A high–molecular weight IκB-Ub conjugate was observed in the absence of TNF-α at 5% (v/v) CO_2_ as previously observed (*35*) (Fig. 5A, arrow, top, and fig. S5). The IκB-Ub conjugate was not observed in the presence of TNF-α at 5% (v/v) CO_2_, consistent with its degradation in the proteasome. Significantly, no high–molecular weight IκB-Ub conjugate was observed in the absence of TNF-α at 10% (v/v) CO_2_. This observation is consistent with a decrease in Ub conjugation to IκB at elevated CO_2_ and the in vitro data of Fig. 3A. The experimental observations were independent of small variations in input Ub and IκB evident across the biological replicates. We permitted the higher Ub input signal level at 10% (v/v) CO_2_ to be certain that the loss of the high–molecular weight IκB-Ub conjugate was not an artefact of a lower input Ub. Note that under the conditions of this experiment, the IκB-Ub conjugate was observed to degrade in the presence of TNF-α at 10% (v/v) CO_2_. Thus, the observation of a change in the formation of an IκB-Ub complex was, by necessity, made in the absence of TNF-α. Future developments will be required to observe changes in IκB-Ub conjugation in the presence of TNF-α.

We assessed the impact of 10% (v/v) CO_2_ on IκB degradation in response to TNF-α. A comparison was made to ambient CO_2_ to increase the likelihood of observing a difference (*25*). HEK 293 cells transfected with Ub WT or Ub K48R were compared for their sensitivity to elevated CO_2_ after stimulation with TNF-α (0.26 ng ml^−1^) and harvesting cells at 30 min (Fig. 5B). We observed that the ratio of the impact of elevated CO_2_ for Ub K48R:Ub WT was consistently <1, indicating that the CO_2_ effect was more significant for Ub WT than Ub K48R.

We further assessed the impact of 10% (v/v) CO_2_ on p65 nuclear localization in response to TNF-α. HEK 293 cells transfected with Ub WT or Ub K48R were compared for their sensitivity to elevated CO_2_ after stimulation with TNF-α (10 ng ml^−1^) and harvesting cells at 30 min (Fig. 5B). We observed that the ratio of the impact of elevated CO_2_ for Ub K48R:Ub WT was also consistently <1, indicating that Ub K48R altered the sensitivity of the experiment to CO_2_. In this case, the result indicates that Ub K48R overexpression increased the sensitivity of p65 nuclear localization to CO_2_. Therefore, it is interesting to note that CO_2_, through Ub K48, might have varying effects at different parts of the NF-κB pathway depending on its local roles. For example, Ub K48 is linked to protein nuclear export (*36*), and these processes might influence p65 nuclear localization and downstream effects at chromatin. However, whatever the impact of CO_2_ on varying parts of the TNF-α response, all with their characteristic response and dynamics, the result is the down-regulation of NF-κB expression.

Considering the observations that Ub binds CO_2_, has its biochemistry altered by CO_2_ in vitro and in vivo and overexpression of a mutant Ub that cannot bind CO_2_ in cellulo, ablates a response to hypercapnia, and alters the CO_2_ response as varying parts of the response pathway, we conclude that Ub is a CO_2_-binding protein.

## DISCUSSION

The sensing of bioactive gases is of fundamental importance to mammalian physiology. Soluble guanylate cyclase is the nitric oxide receptor (*37*), while oxygen sensing is achieved by PHD1-3 catalyzed prolyl hydroxylation of hypoxia-inducible factors (HIF-1α, HIF-2α, and HIF-3α), which facilitates HIF-α regulation in the PHD1-3–HIF-α–pVHL signaling axis (*38*, *39*). Analysis of transcriptional responses to elevated CO_2_ in *Drosophila* identified up-regulated gene ontology (GO) families relating to metabolic functions. In contrast, most of the down-regulated GO families had either immune- or fertility-related annotations (*40*). Similar experiments in *Caenorhabditis elegans* identified 488 up- or down-regulated genes after 1-hour exposure to elevated CO_2_ (*41*). In addition to transcription responses, physiological responses to hypercapnia in *Drosophil*a included altered embryo morphogenesis, egg laying, egg hatching, and innate immune responses. *C. elegans* responses included altered body muscle organization, slowed development, reduced fertility, and increased life span. No candidate CO_2_-binding protein that might explain its various physiological effects has been identified to date. Ub fulfils the criteria of a CO_2_-binding protein and might explain the diverse physiological impact of hypercapnia.

Elevated CO_2_ is reasonably well tolerated in mammals, while Ub conjugation is an essential cellular function. Therefore, it is significant that carbamate formation on Ub across the range of 0 to 3 mM CO_2_ reduces Ub conjugation at K48 by just over 10%. Such a reduction in Ub conjugation may be sufficient to have a physiological impact, but not too significant an effect of being lethal. The specific carbamate formation sites on Ub also guide future avenues of investigation for molecular responses to CO_2_. Carbamate formation at Ub K48 suggests an influence on signaling pathways regulated by proteasomal degradation, as evidenced here for NF-κB–dependent transcription. We cannot exclude the possibility that carbamylation at K48 (or another site) acts to expand the Ub code in the presence of conjugates at other sites (*42*). Carbamate formation at Ub K6 and K33 suggests a mechanism by which hypercapnia might influence mitophagy/xenophagy and post-Golgi membrane protein trafficking, respectively (*16*, *43*). Also, of note, Ub N-terminal carbamylation might be physiologically important because the head-to-tail linked (also known as “linear”) Ub chains also regulate immune signaling (*44*). The impacts of carbamylation upon Ub polymerization in vitro and in vivo at these sites await future investigation. No motif identifying the propensity of a site to carbamylation is evident, most likely because carbamylation depends on a structurally privileged local environment that lowers local p*K*_aH_, rather than a defined primary motif. Carbamylation in Ub-like molecules (e.g., prokaryotic Ub-like protein) will therefore require future experiments and is not currently amenable to prediction.

The identification of Ub as a CO_2_-binding protein might also decipher conflicting data surrounding the identity(ies) of the site(s) of action of CO_2_ along the NF-κB pathway. Several studies suggest that CO_2_ affects the canonical NF-κB pathway components, including IκB-α (*45*) and the noncanonical pathway (*25*, *46*, *47*). In the canonical pathway, in addition to IκB, the E3 ligase cIAP1 and RIPK1 serine-threonine kinase at the TNF receptor complex require Ub K48-linked poly-Ub for pathway activation (*48*). In the noncanonical pathway, ubiquitination with K48-linked poly-Ub of TRAF2 by cIAP1 and of p100 for its processing to p52 is required for activation (*49*). Ub is the common entity that could underpin explanations as to why both pathways are sensitive to CO_2_. The future selection of other ligands will allow this to be selectively probed.

In summary, Ub is a CO_2_-binding protein through carbamate formation. Physiological levels of CO_2_ regulate Ub conjugation at K48 in vitro, and overexpression of a Ub mutant that is unable to conjugate at the K48 CO_2_-dependent site ablates a CO_2_-dependent phenotype in cellulo. On the basis of our findings, we postulate that the regulation of ubiquitination by CO_2_ explains one of hypercapnia’s broad physiological effects.

## MATERIALS AND METHODS

### CO_2_ trapping

All CO_2_-trapping experiments were carried out with recombinant protein (500 μg) in phosphate buffer (3 ml, 50 mM, pH 7.4). This solution was transferred to a potentiometric titrator (902 Titrando; Metrohm) and incubated at 25°C with stirring. A freshly made solution of TEO tetrafluoroborate (Et_3_OBF_4_; 280 mg, 1.47 mmol) in phosphate buffer (1 ml) was added stepwise with a constant pH being maintained (pH 7.4) through the slow addition of 1 M NaOH solution via the automatic burette. The reaction mixture was stirred, with the pH being maintained, for 1 hour after the final Et_3_OBF4 addition to ensure that all TEO was hydrolyzed. The reaction mixture was then dialyzed against dH_2_O (1 liter) overnight.

### Mass spectrometry

The water was removed from a postdialysis-trapped sample supernatant using a centrifugal vacuum concentrator. Protein was resuspended in urea solution (8 M, 500 μl), and disulfide bonds in the sample were reduced using dithiothreitol (DTT; 25 mM final concentration) at 37°C for 1 hour. The resulting free thiol groups were alkylated using iodoacetamide (40 mM) in the dark for 1 hour at room temperature. The sample was diluted to 1 M urea with ammonium bicarbonate buffer and digested with trypsin gold [mass spectrometry grade, Promega; 1:25 (w/w) ratio to protein] overnight at 37°C. The solution of digested proteins was desalted and resolved using a C18 column (ZipTip, Merck Millipore), dried down, and resuspended in 4% (v/v) acetonitrile and 0.05% (v/v) trifluoroacetic acid. The eluted peptides were analyzed by LC-MS-MS on a QStar Pulsar QTOF mass spectrometer (Sciex) coupled to nano-LC instrument. Peptides were eluted from an LC gradient from 3 to 80% (v/v) acetonitrile and injected online to the mass spectrometer [information dependent acquisition (IDA) mode, mass range of 300 to 1600 Da, MS accumulation time of 1 s, ion source voltage of 2300 V, three MS-MS spectra per cycle, MS-MS mass range of 100 to 1600 Da, and MS-MS accumulation time of 3 s]. The post–ESI-MS-MS raw data files were converted into .mgf files using the freeware MSConvert provided by ProteoWizard (*50*) and analyzed using PEAKS Studio 10.5 software (*51*) including the variable modifications ethylation (28.0313 Da at D or E), carboxyethylation [72.0211 for ^12^CO_2_ or 73.0211 for ^13^CO_2_ at K or protein N-terminal groups], oxidation (M), acetylation (N terminus), and the fixed modification carbimidomethyl (C). These data were then refined using a false discovery rate of 1% and a PTM AScore of 50.

### NMR spectroscopy

NMR experiments were performed on a Bruker Avance-III 800 MHz spectrometer equipped with a QCI cryoprobe. All carbamylation experiments were performed at 37°C in a buffer containing 20 mM sodium phosphate, 0.02% (w/v) NaN_3_, and 10% (v/v) D_2_O at pH 7.4. The buffer was prepared with 100 mM NaH^13^CO_3_ unless otherwise indicated. ^13^C spectra were acquired with a 45° pulse, spectral width of 12820.5 Hz, an acquisition time of 847 ms for 21,738 points, and a recycle delay of 2.5 s. Spectra with ^13^C- and ^15^N-labeled Ub were acquired with ^15^N decoupling during an 80-ms acquisition period. All processing was performed with Mestrelab MNova within an NMRBox (*52*) environment. Ub Lys p*K*_aH_ values were determined by following either N_ζ_ or C_ε_/C_α_ chemical shifts measured via triple-resonance H_ε_2C_ε_N_ζ_ experiments (H2CN) (*53*) or ^1^H-^13^C HSQC experiment, respectively, within the pH range from 8.6 to 12.3 at 23°C. Initially, ^13^C/^15^N Ub was exchanged into 5 mM N-cyclohexyl-2-aminoethanesulfonic acid (CHES)/5 mM N-cyclohexyl-3-aminopropanesulfonic acid (CAPS) buffer at pH 10.5 and separated into two 500-μl 1 mM Ub aliquots that were run as two divergent samples for lower or higher pH measurements. After each experiment, these samples were consecutively exchanged into the same buffer with either increasing or decreasing pH by the addition of 0.1 M HCl and 0.1 M KOH in approximately 0.3 (H2CN) or 0.4 (HSQC) pH unit steps. D_2_O was added to each sample to a final concentration of 5% (v/v). The p*K*_aH_ values were obtained by fitting the observed signal shifts to a Henderson-Hasselbalch model. The errors in reported p*K*_aH_ values (Table 1) for each residue were assessed by fitting 2000 Monte Carlo–generated synthetic datasets, in which Gaussian noise was added to the chemical shifts according to the resolution in the respective H2CN or ^1^H-^13^C HSQC spectra.

### Recombinant protein production

WT human Ub derived from pET15 (*54*) was cloned into the Nde I and Bam HI restriction sites of pET28a with a stop codon introduced to preclude expression of the His_6_ affinity tag. Mutant human Ub–expressing constructs were derived from the WT plasmid by site-directed mutagenesis. Recombinant WT and mutant Ub were expressed as untagged proteins from pET28a in *E. coli* BL21(DE3) at 20°C for 16 hours with 0.4 mM isopropyl-β-d-thiogalactoside (IPTG). Pelleted bacteria (10 ml) were suspended in sonication buffer (phosphate-buffered saline, 50 ml) including SIGMAFAST Protease Inhibitor Cocktail Tablets, lysed by sonication (180 s on ice), and centrifuged (50,000*g*, 40 min, 4°C). The supernatant was incubated on ice with the addition of 70% (w/v) perchloric acid with vigorous stirring until solution pH dropped to 4.5. The solution was left to stir for 1 hour and then centrifuged to remove precipitate (5000*g*, 40 min, 4°C). The remaining supernatant was dialyzed against 50 mM ammonium acetate buffer overnight (1 liter, 4°C). Sample was centrifuged (50,000*g*, 40 min, 4°C), and supernatant was dialyzed against purification buffer for 8 hours (10 mM tris, pH 7.6). Protein from this sample was then purified using size exclusion chromatography (Superdex 75).

mE1 was expressed from pET28a in *E. coli* BL21(DE3) at 16°C for 20 hours with 0.5 mM IPTG (*55*). Pelleted bacteria (10 ml) were suspended in sonication buffer [50 ml; 50 mM tris-HCl (pH 8), 150 mM NaCl, 0.1% (v/v) Triton X-100, 1 mM EDTA, 1 mM DTT, phenylmethylsulfonyl fluoride (PMSF; 0.1 mg/ml)], lysed by sonication (180 s on ice), and centrifuged (50,000*g*, 40 min, 4°C). Protein was affinity-purified from the supernatant using a 5-ml HisPrep HP Ni-NTA column (GE Healthcare) on an AKTA Pure chromatography system at 2 ml min^−1^ (GE Healthcare). Eluted protein was concentrated and buffer-exchanged with 10 mM tris-HCl (pH 8), 1 mM EDTA, and 1 mM DTT before additional purification by ion exchange chromatography and size exclusion chromatography at 0.5 ml min^−1^.

E2-25K and Ubc13 were expressed from pGEX-4T in *E. coli* BL21(DE3) at 16°C for 20 hours with 0.5 mM IPTG (*56*, *57*). Pelleted bacteria (10 ml) were suspended in sonication buffer [50 ml; 50 mM tris-HCl (pH 8), 750 mM NaCl, 1% (v/v) NP-40], lysed by sonication (180 s on ice), and centrifuged (50,000*g*, 40 min, 4°C). Protein was affinity-purified from the supernatant using a 5-ml glutathione *S*-transferase (GST)–agarose column (Thermo Fisher Scientific) on an AKTA Pure chromatography system at 1 ml min^−1^ (GE Healthcare).

pET28a-UEV1 was a gift from Cheryl Arrowsmith (Addgene plasmid #25619; http://n2t.net/addgene:25619; RRID:Addgene_25619). UEV1 was expressed from pET28a in *E. coli* BL21(DE3) at 16°C for 20 hours with 0.5 mM IPTG. Pelleted bacteria (10 ml) were suspended in sonication buffer [50 ml; 20 mM tris 8.0, 10% (v/v) glycerol, 300 mM NaCl, 2 mM 2-mercaptoethanol, 0.2 mM tris(2-carboxyethyl)phosphine, 0.1% (v/v) Triton X-100, and 2 mM PMSF]. Protein was affinity-purified from the supernatant using a 5-ml HisPrep HP Ni-NTA column (GE Healthcare) on an AKTA Pure chromatography system at 2 ml min^−1^ (GE Healthcare). The His_6_ affinity tag was removed using tobacco etch virus (TEV) protease (Sigma-Aldrich), and the protein was passed over a second Ni-NTA column. Protein from this sample was then purified by size exclusion chromatography (Superdex 75).

### Ub conjugation assays

Conjugation assays were performed with 0.2 mM Ub, 2.5 mM adenosine 5′-triphosphate (ATP), 2.5 mM MgCl_2_, and 0.5 mM DTT in 250 mM tris-HCl (pH 7.5). Conjugation reactions at Ub K48 included 0.1 μM mE1 and 20 μM E2-25K (also known as Ube2k). Conjugation at Ub K63 included 1 μM mE1, 8 μM Ubc13, and 8 μM UEV1. Experiments were performed from 0 to 50 mM CO_2_/HCO_3_^−^ with [anion] maintained at 50 mM with supplementary Cl^−^. Reactions were performed in 50-μl volumes at 37°C for 30 min and started with mE1 addition. Reactions were stopped with 1× Laemmli buffer and electrophoresed by SDS–polyacrylamide gel electrophoresis for imaging and quantification using ImageJ (*58*).

### Western blotting

Polyacrylamide gels [1.0 mm; 10% (v/v) bis-acrylamide resolving and 5% (v/v) bis-acrylamide stacking] were poured using the Mini-PROTEAN Tetra Electrophoresis System. Samples were mixed 1:1 (v:v) with loading buffer [50 mM tris-HCl (pH 6.8), 2% (w/v) SDS, 0.1% (w/v) bromophenol blue, 10% (v/v) glycerol, and 100 mM DTT], incubated at 95°C for 5 min, and run at 20 V cm^−1^ in running buffer [25 mM tris-HCl (pH 6.8), 200 mM glycine, 0.1% (w/v) SDS]. Proteins were transferred at 2 V cm^−1^ at 4°C overnight in transfer buffer [25 mM tris-HCl (pH 8.5), 190 mM glycine, and 15% (v/v) methanol]. Membranes were washed for 5 min in TBS-T [25 mM tris-HCl (pH 7.5), 150 mM NaCl, 0.05% (v/v) Tween 20] and incubated in blocking buffer [5% (w/v) nonfat milk in TBS-T] for 2 hours at room temperature. Membranes were washed three times in TBS-T for 10 min each and then probed with primary antibody diluted in blocking buffer. Membranes were washed again with TBS-T and then probed with secondary antibody diluted in blocking buffer. Membranes were again washed with TBS-T before developing with ECL Western Blotting Detection Reagent at room temperature. Blots were imaged and quantified using ImageJ (*58*). The signal for the α-HA (human influenza hemagglutinin) antibody used for the quantitative Western blot of Fig. 4C was confirmed to lie on a straight line of a plot of Western blot signal versus loaded protein lysate for which the slope was significantly nonzero (fig. S6). The conditions of the Western blot were therefore suitable for quantitation.

### GFP reporter assay

NF-κB/293/GFP-Luc cells (System Biosciences) were cultured until 80% confluency in Dulbecco’s modified Eagle’s medium (DMEM) supplemented with 10% (v/v) heat-inactivated newborn calf serum, 100 μM nonessential amino acids, 50 U of penicillin, and 50 μg of streptomycin. Cells were passaged into 24-well plates and allowed to adhere for 6 hours before transfection. Transfections were performed using Lipofectamine 3000 (Invitrogen) in Opti-MEM media (Gibco) with 1 μg of DNA per well. Human Ub WT and K48R were expressed from pRK5 (*60*). A plasmid expressing human Ub K63R was generated from the WT template by site-directed mutagenesis. Cells were incubated at 37°C and 5% (v/v) CO_2_ for 20 hours, and then the medium was exchanged for DMEM without phenol red containing 25 mM Hepes buffer (pH 7.4) supplemented with 10% (v/v) heat-inactivated newborn calf serum, 100 μM nonessential amino acids, and 25 mM or 35 mM sodium bicarbonate. Cells were incubated at 37°C at 5 or 10% (v/v) CO_2_, respectively, with TNF-α (30 ng ml^−1^) for 20 hours before lysis with M-PER extraction reagent and fluorescence counting in a microplate reader.

### Immunoprecipitation

Antibody immunoprecipitation columns were produced using the Pierce Coimmunoprecipitation Kit (Thermo Fisher Scientific). Briefly, aminolink coupling resin was washed with coupling buffer and incubated with 70 μg of α-Ub antibody (Abcam ab134953) and sodium cyanoborohydride for 2 hours at room temperature with mixing. The column was washed and incubated with quenching buffer and sodium cyanoborohydride for 30 min with mixing. The column was washed and stored at 4°C before immediate use. Cells were incubated at 37°C with 5 or 10% (v/v) CO_2_, respectively, with TNF-α (30 ng ml^−1^) for 20 hours before lysis with M-PER extraction reagent (Thermo Fisher Scientific, 78501). Input Western blots were carried out as described using 5 μg of total cell lysate and probed with both α-Ub and α-IκB antibodies. Input material for immunoprecipitation was normalized to total protein assessed by Bradford assay. Cell lysate from the same experiment was incubated with the α-Ub resin overnight at 4°C with rolling. Columns were centrifuged at 500*g* for 30 s to remove buffer, samples were eluted with 50 μl of elution buffer (pH 2.8), and the pH was neutralized with the addition of 1 M tris (pH 9.5). Eluted samples were then probed using an α-IκB antibody and the described Western blot protocol.

### p65 assay

NF-κB nuclear p65 was assayed by enzyme-linked immunosorbent assay (ELISA) (abcam133112) according to the manufacturer’s instructions.

## Supplementary Materials

### This PDF file includes:

Figs. S1 to S6 Legends for Raw data

### Other Supplementary Material for this manuscript includes the following:

Raw data

## Notes

View/request a protocol for this paper from *Bio-protocol*.

## References

[1] J. Chandrashekar, D. Yarmolinsky, L. von Buchholtz, Y. Oka, W. Sly, N. J. P. Ryba, C. S. Zuker, The taste of carbonation. Science 326, 443–445 (2009). 19833970 10.1126/science.1174601 PMC3654389

[2] E. P. Cummins, A. C. Selfridge, P. H. Sporn, J. I. Sznajder, C. T. Taylor, Carbon dioxide-sensing in organisms and its implications for human disease. Cell. Mol. Life Sci. 71, 831–845 (2014). 24045706 10.1007/s00018-013-1470-6 PMC3945669

[3] P. D. Townsend, P. M. Holliday, S. Fenyk, K. C. Hess, M. A. Gray, D. R. W. Hodgson, M. J. Cann, Stimulation of mammalian G-protein-responsive adenylyl cyclases by carbon dioxide. J. Biol. Chem. 284, 784–791 (2009). 19008230 10.1074/jbc.M807239200 PMC2613629

[4] S. V. Wiggins, C. Steegborn, L. R. Levin, J. Buck, Pharmacological modulation of the CO2/HCO3−/pH-, calcium-, and ATP-sensing soluble adenylyl cyclase. Pharmacol. Ther. 190, 173–186 (2018). 29807057 10.1016/j.pharmthera.2018.05.008 PMC6484840

[5] Y. Chen, M. J. Cann, T. N. Litvin, V. Iourgenko, M. L. Sinclair, L. R. Levin, J. Buck, Soluble adenylyl cyclase as an evolutionarily conserved bicarbonate sensor. Science 289, 625–628 (2000). 10915626 10.1126/science.289.5479.625

[6] L. Meigh, S. A. Greenhalgh, T. L. Rodgers, M. J. Cann, D. I. Roper, N. Dale, CO2 directly modulates connexin 26 by formation of carbamate bridges between subunits. eLife 2, e01213 (2013). 24220509 10.7554/eLife.01213 PMC3821526

[7] Y. Zhou, L. A. Skelton, L. Xu, M. P. Chandler, J. M. Berthiaume, W. F. Boron, Role of receptor protein tyrosine phosphataseγin sensing extracellular CO2and HCO3−. J. Am. Soc. Nephrol. 27, 2616–2621 (2016). 26839367 10.1681/ASN.2015040439 PMC5004642

[8] G. H. Lorimer, H. M. Miziorko, Carbamate formation on the epsilon-amino group of a lysyl residue as the basis for the activation of ribulosebisphosphate carboxylase by CO2 and Mg2+. Biochemistry 19, 5321–5328 (1980). 6778504 10.1021/bi00564a027

[9] J. B. Matthew, R. J. Wittebort, D. F. Hayes, T. M. Rothgeb, R. S. Gurd, F. R. N. Gurd, Reaction of carbon-dioxide with amino-groups in model systems, peptide hormones, and hemoglobin. Abstr. Pap. Am. Chem. Soc. 174, 92 (1977).

[10] V. L. Linthwaite, J. M. Janus, A. P. Brown, D. Wong-Pascua, A. M. C. O’Donoghue, A. Porter, A. Treumann, D. R. W. Hodgson, M. J. Cann, The identification of carbon dioxide mediated protein post-translational modifications. Nat. Commun. 9, 3092 (2018). 30082797 10.1038/s41467-018-05475-z PMC6078960

[11] G. H. Lorimer, Carbon dioxide and carbamate formation: The makings of a biochemical control system. Trends Biochem. Sci. 8, 65–68 (1983).

[12] D. Komander, The emerging complexity of protein ubiquitination. Biochem. Soc. Trans. 37, 937–953 (2009). 19754430 10.1042/BST0370937

[13] F. Ohtake, H. Tsuchiya, Y. Saeki, K. Tanaka, K63 ubiquitylation triggers proteasomal degradation by seeding branched ubiquitin chains. Proc. Natl. Acad. Sci. U.S.A. 115, E1401–E1408 (2018). 29378950 10.1073/pnas.1716673115 PMC5816176

[14] W. Li, Y. Ye, Polyubiquitin chains: Functions, structures, and mechanisms. Cell. Mol. Life Sci. 65, 2397–2406 (2008). 18438605 10.1007/s00018-008-8090-6 PMC2700825

[15] J. Callis, J. A. Raasch, R. D. Vierstra, Ubiquitin extension proteins of Arabidopsis thaliana. Structure, localization, and expression of their promoters in transgenic tobacco. J. Biol. Chem. 265, 12486–12493 (1990). 2165066

[16] K. N. Swatek, D. Komander, Ubiquitin modifications. Cell Res. 26, 399–422 (2016). 27012465 10.1038/cr.2016.39 PMC4822133

[17] K. Iwai, Diverse roles of the ubiquitin system in NF-κB activation. Biochim. Biophys. Acta 1843, 129–136 (2014). 23523932 10.1016/j.bbamcr.2013.03.011

[18] J. S. Morrow, P. Keim, F. R. Gurd, CO2 adducts of certain amino acids, peptides, and sperm whale myoglobin studied by carbon 13 and proton nuclear magnetic resonance. J. Biol. Chem. 249, 7484–7494 (1974). 4436319

[19] T. R. Cundari, A. K. Wilson, M. L. Drummond, H. E. Gonzalez, K. R. Jorgensen, S. Payne, J. Braunfeld, M. de Jesus, V. M. Johnson, CO_2_-formatics: How do proteins bind carbon dioxide? J. Chem. Inf. Model. 49, 2111–2115 (2009). 19705826 10.1021/ci9002377

[20] S. Y. Lee, Y. S. Choi, E. H. Kim, H. K. Cheong, Y. J. Lee, J. G. Lee, Y. Ye, K. S. Ryu, Nonenzymatic acetylation of ubiquitin Lys side chains is modulated by their neighboring residues. FEBS J. 285, 1277–1289 (2018). 29430834 10.1111/febs.14404 PMC5947880

[21] L. Buetow, D. T. Huang, Structural insights into the catalysis and regulation of E3 ubiquitin ligases. Nat. Rev. Mol. Cell Biol. 17, 626–642 (2016). 27485899 10.1038/nrm.2016.91 PMC6211636

[22] G. L. Grice, J. A. Nathan, The recognition of ubiquitinated proteins by the proteasome. Cell. Mol. Life Sci. 73, 3497–3506 (2016). 27137187 10.1007/s00018-016-2255-5 PMC4980412

[23] S. Faggiano, C. Alfano, A. Pastore, The missing links to link ubiquitin: Methods for the enzymatic production of polyubiquitin chains. Anal. Biochem. 492, 82–90 (2016). 26470940 10.1016/j.ab.2015.09.013

[24] N. Nin, A. Muriel, O. Peñuelas, L. Brochard, J. A. Lorente, N. D. Ferguson, K. Raymondos, F. Ríos, D. A. Violi, A. W. Thille, M. González, A. J. Villagomez, J. Hurtado, A. R. Davies, B. Du, S. M. Maggiore, L. Soto, G. D’Empaire, D. Matamis, F. Abroug, R. P. Moreno, M. A. Soares, Y. Arabi, F. Sandi, M. Jibaja, P. Amin, Y. Koh, M. A. Kuiper, H.-H. Bülow, A. A. Zeggwagh, A. Anzueto, J. I. Sznajder, A. Esteban; VENTILA Group, Severe hypercapnia and outcome of mechanically ventilated patients with moderate or severe acute respiratory distress syndrome. Intensive Care Med. 43, 200–208 (2017). 28108768 10.1007/s00134-016-4611-1 PMC5630225

[25] E. P. Cummins, K. M. Oliver, C. R. Lenihan, S. F. Fitzpatrick, U. Bruning, C. C. Scholz, C. Slattery, M. O. Leonard, P. McLoughlin, C. T. Taylor, NF-κB links CO2 sensing to innate immunity and inflammation in mammalian cells. J. Immunol. 185, 4439–4445 (2010). 20817876 10.4049/jimmunol.1000701

[26] D. O’Toole, P. Hassett, M. Contreras, B. D. Higgins, S. T. W. McKeown, D. F. McAuley, T. O’Brien, J. G. Laffey, Hypercapnic acidosis attenuates pulmonary epithelial wound repair by an NF- B dependent mechanism. Thorax 64, 976–982 (2009). 19617214 10.1136/thx.2008.110304

[27] K. Takeshita, Y. Suzuki, K. Nishio, O. Takeuchi, K. Toda, H. Kudo, N. Miyao, M. Ishii, N. Sato, K. Naoki, T. Aoki, K. Suzuki, R. Hiraoka, K. Yamaguchi, Hypercapnic acidosis attenuates endotoxin-induced nuclear factor-[kappa]B activation. Am. J. Respir. Cell Mol. Biol. 29, 124–132 (2003). 12600832 10.1165/rcmb.2002-0126OC

[28] M. Shigemura, E. Lecuona, J. I. Sznajder, Effects of hypercapnia on the lung. J. Physiol. 595, 2431–2437 (2017). 28044311 10.1113/JP273781 PMC5390878

[29] J. G. Laffey, D. O’Croinin, P. McLoughlin, B. P. Kavanagh, Permissive hypercapnia--role in protective lung ventilatory strategies. Intensive Care Med. 30, 347–356 (2004). 14722644 10.1007/s00134-003-2051-1

[30] N. Nin, M. Angulo, A. Briva, Effects of hypercapnia in acute respiratory distress syndrome. Ann. Transl. Med. 6, 37 (2018). 29430454 10.21037/atm.2018.01.09 PMC5799147

[31] D. M. Chudakov, M. V. Matz, S. Lukyanov, K. A. Lukyanov, Fluorescent proteins and their applications in imaging living cells and tissues. Physiol. Rev. 90, 1103–1163 (2010). 20664080 10.1152/physrev.00038.2009

[32] A. C. Selfridge, M. A. S. Cavadas, C. C. Scholz, E. L. Campbell, L. C. Welch, E. Lecuona, S. P. Colgan, K. E. Barrett, P. H. S. Sporn, J. I. Sznajder, E. P. Cummins, C. T. Taylor, Hypercapnia suppresses the HIF-dependent adaptive response to hypoxia. J. Biol. Chem. 291, 11800–11808 (2016). 27044749 10.1074/jbc.M116.713941 PMC4882447

[33] Z. C. Cook, M. A. Gray, M. J. Cann, Elevated carbon dioxide blunts mammalian cAMP signaling dependent on inositol 1,4,5-triphosphate receptor-mediated Ca2+ release. J. Biol. Chem. 287, 26291–26301 (2012). 22654111 10.1074/jbc.M112.349191 PMC3406713

[34] K. Lang, C. Wagner, G. Haddad, O. Burnekova, J. Geibel, Intracellular pH activates membrane-bound Na(+)/H(+) exchanger and vacuolar H(+)-ATPase in human embryonic kidney (HEK) cells. Cell. Physiol. Biochem. 13, 257–262 (2003). 14586169 10.1159/000074540

[35] Y. Tsuchiya, T. Asano, K. Nakayama, T. Kato Jr., M. Karin, H. Kamata, Nuclear IKKbeta is an adaptor protein for IkappaBalpha ubiquitination and degradation in UV-induced NF-kappaB activation. Mol. Cell 39, 570–582 (2010). 20797629 10.1016/j.molcel.2010.07.030

[36] S. Hirayama, M. Sugihara, D. Morito, S. I. Iemura, T. Natsume, S. Murata, K. Nagata, Nuclear export of ubiquitinated proteins via the UBIN-POST system. Proc. Natl. Acad. Sci. U.S.A. 115, E4199–E4208 (2018). 29666234 10.1073/pnas.1711017115 PMC5939056

[37] W. P. Arnold, C. K. Mittal, S. Katsuki, F. Murad, Nitric oxide activates guanylate cyclase and increases guanosine 3′:5′-cyclic monophosphate levels in various tissue preparations. Proc. Natl. Acad. Sci. U.S.A. 74, 3203–3207 (1977). 20623 10.1073/pnas.74.8.3203 PMC431498

[38] G. L. Wang, B. H. Jiang, E. A. Rue, G. L. Semenza, Hypoxia-inducible factor 1 is a basic-helix-loop-helix-PAS heterodimer regulated by cellular O2 tension. Proc. Natl. Acad. Sci. U.S.A. 92, 5510–5514 (1995). 7539918 10.1073/pnas.92.12.5510 PMC41725

[39] D. R. Mole, P. H. Maxwell, C. W. Pugh, P. J. Ratcliffe, Regulation of HIF by the von Hippel-Lindau tumour suppressor: Implications for cellular oxygen sensing. IUBMB Life 52, 43–47 (2001). 11795592 10.1080/15216540252774757

[40] I. T. Helenius, T. Krupinski, D. W. Turnbull, Y. Gruenbaum, N. Silverman, E. A. Johnson, P. H. S. Sporn, J. I. Sznajder, G. J. Beitel, Elevated CO2 suppresses specific Drosophila innate immune responses and resistance to bacterial infection. Proc. Natl. Acad. Sci. U.S.A. 106, 18710–18715 (2009). 19846771 10.1073/pnas.0905925106 PMC2773965

[41] K. Sharabi, A. Hurwitz, A. J. Simon, G. J. Beitel, R. I. Morimoto, G. Rechavi, J. I. Sznajder, Y. Gruenbaum, Elevated CO2 levels affect development, motility, and fertility and extend life span in Caenorhabditis elegans. Proc. Natl. Acad. Sci. U.S.A. 106, 4024–4029 (2009). 19237558 10.1073/pnas.0900309106 PMC2656198

[42] D. Komander, M. Rape, The ubiquitin code. Annu. Rev. Biochem. 81, 203–229 (2012). 22524316 10.1146/annurev-biochem-060310-170328

[43] W. C. Yuan, Y. R. Lee, S. Y. Lin, L. Y. Chang, Y. P. Tan, C. C. Hung, J. C. Kuo, C. H. Liu, M. Y. Lin, M. Xu, Z. J. Chen, R. H. Chen, K33-linked polyubiquitination of coronin 7 by Cul3-KLHL20 ubiquitin E3 ligase regulates protein trafficking. Mol. Cell 54, 586–600 (2014). 24768539 10.1016/j.molcel.2014.03.035

[44] B. Gerlach, S. M. Cordier, A. C. Schmukle, C. H. Emmerich, E. Rieser, T. L. Haas, A. I. Webb, J. A. Rickard, H. Anderton, W. W. L. Wong, U. Nachbur, L. Gangoda, U. Warnken, A. W. Purcell, J. Silke, H. Walczak, Linear ubiquitination prevents inflammation and regulates immune signalling. Nature 471, 591–596 (2011). 21455173 10.1038/nature09816

[45] C. Masterson, D. O’Toole, A. Leo, P. McHale, S. Horie, J. Devaney, J. G. Laffey, Effects and mechanisms by which hypercapnic acidosis inhibits sepsis-induced canonical nuclear factor-κB signaling in the lung. Crit. Care Med. 44, e207–e217 (2016). 26584194 10.1097/CCM.0000000000001376

[46] K. M. Oliver, C. R. Lenihan, U. Bruning, A. Cheong, J. G. Laffey, P. McLoughlin, C. T. Taylor, E. P. Cummins, Hypercapnia induces cleavage and nuclear localization of RelB protein, giving insight into CO2 sensing and signaling. J. Biol. Chem. 287, 14004–14011 (2012). 22396550 10.1074/jbc.M112.347971 PMC3340129

[47] C. E. Keogh, C. C. Scholz, J. Rodriguez, A. C. Selfridge, A. von Kriegsheim, E. P. Cummins, Carbon dioxide-dependent regulation of NF-κB family members RelB and p100 gives molecular insight into CO2-dependent immune regulation. J. Biol. Chem. 292, 11561–11571 (2017). 28507099 10.1074/jbc.M116.755090 PMC5500817

[48] F. Ikeda, Linear ubiquitination signals in adaptive immune responses. Immunol. Rev. 266, 222–236 (2015). 26085218 10.1111/imr.12300 PMC4506786

[49] A. Borghi, L. Verstrepen, R. Beyaert, TRAF2 multitasking in TNF receptor-induced signaling to NF-κB, MAP kinases and cell death. Biochem. Pharmacol. 116, 1–10 (2016). 26993379 10.1016/j.bcp.2016.03.009

[50] J. D. Holman, D. L. Tabb, P. Mallick, Employing ProteoWizard to convert raw mass spectrometry data. Curr. Protoc. Bioinformatics 46, 11–19 (2014). 10.1002/0471250953.bi1324s46 PMC4113728 24939128

[51] B. Ma, K. Zhang, C. Hendrie, C. Liang, M. Li, A. Doherty-Kirby, G. Lajoie, PEAKS: Powerful software for peptide de novo sequencing by tandem mass spectrometry. Rapid Commun. Mass Spectrom. 17, 2337–2342 (2003). 14558135 10.1002/rcm.1196

[52] M. W. Maciejewski, A. D. Schuyler, M. R. Gryk, I. I. Moraru, P. R. Romero, E. L. Ulrich, H. R. Eghbalnia, M. Livny, F. Delaglio, J. C. Hoch, NMRbox: A resource for biomolecular NMR computation. Biophys. J. 112, 1529–1534 (2017). 28445744 10.1016/j.bpj.2017.03.011 PMC5406371

[53] I. Andre, S. Linse, F. A. Mulder, Residue-specific pKa determination of lysine and arginine side chains by indirect 15N and 13C NMR spectroscopy: Application to apo calmodulin. J. Am. Chem. Soc. 129, 15805–15813 (2007). 18044888 10.1021/ja0721824

[54] P. S. Brzovic, A. Lissounov, D. E. Christensen, D. W. Hoyt, R. E. Klevit, A UbcH5/ubiquitin noncovalent complex is required for processive BRCA1-directed ubiquitination. Mol. Cell 21, 873–880 (2006). 16543155 10.1016/j.molcel.2006.02.008

[55] A. F. Carvalho, M. P. Pinto, C. P. Grou, R. Vitorino, P. Domingues, F. Yamao, C. Sá-Miranda, J. E. Azevedo, High-yield expression in Escherichia coli and purification of mouse ubiquitin-activating enzyme E1. Mol. Biotechnol. 51, 254–261 (2012). 22012022 10.1007/s12033-011-9463-x

[56] A. Pichler, P. Knipscheer, E. Oberhofer, W. J. van Dijk, R. Körner, J. V. Olsen, S. Jentsch, F. Melchior, T. K. Sixma, SUMO modification of the ubiquitin-conjugating enzyme E2-25K. Nat. Struct. Mol. Biol. 12, 264–269 (2005). 15723079 10.1038/nsmb903

[57] S. Raasi, C. M. Pickart, Ubiquitin chain synthesis. Methods Mol. Biol. 301, 47–55 (2005). 15917625 10.1385/1-59259-895-1:047

[58] V. Girish, A. Vijayalakshmi, Affordable image analysis using NIH Image/ImageJ. Indian J. Cancer 41, 47 (2004). 15105580

[59] Z. Liu, W. P. Zhang, Q. Xing, X. Ren, M. Liu, C. Tang, Noncovalent dimerization of ubiquitin. Angew. Chem. Int. Ed. Eng. 51, 469–472 (2012). 10.1002/anie.201106190 PMC3303887 22109817

[60] K. L. Lim, K. C. M. Chew, J. M. M. Tan, C. Wang, K. K. K. Chung, Y. Zhang, Y. Tanaka, W. Smith, S. Engelender, C. A. Ross, V. L. Dawson, T. M. Dawson, Parkin mediates nonclassical, proteasomal-independent ubiquitination of synphilin-1:Implications for Lewy body formation. J. Neurosci. 25, 2002–2009 (2005). 15728840 10.1523/JNEUROSCI.4474-04.2005 PMC6726069

