# Supplementary material for: Ubiquitin is a carbon dioxide–binding protein
Source: Sci Adv. 2021 Sep 24;7(39):eabi5507. doi: 10.1126/sciadv.abi5507 (PMC8462908; doi:10.1126/sciadv.abi5507)
Supplement: Supplementary file 1 — Figs. S1 to S6 Legends for Raw data [file sciadv.abi5507_sm.pdf]

Science Advances
AAAS

Supplementary Materials for

Ubiquitin is a carbon dioxide–binding protein

Victoria L. Linthwaite, Wes Pawloski, Hamish B. Pegg, Philip D. Townsend, Michael J. Thomas, Victor K. H. So, Adrian P. Brown, David R. W. Hodgson, George H. Lorimer, David Fushman*, Martin J. Cann*

*Corresponding author. Email: fushman@umd.edu (D.F.); m.j.cann@durham.ac.uk (M.J.C.)

Published 24 September 2021, *Sci. Adv.* **7**, eabi5507 (2021)
DOI: 10.1126/sciadv. abi5507

The PDF file includes:

Figs. S1 to S6
Legends for Raw data

Other Supplementary Material for this manuscript includes the following:

Raw data

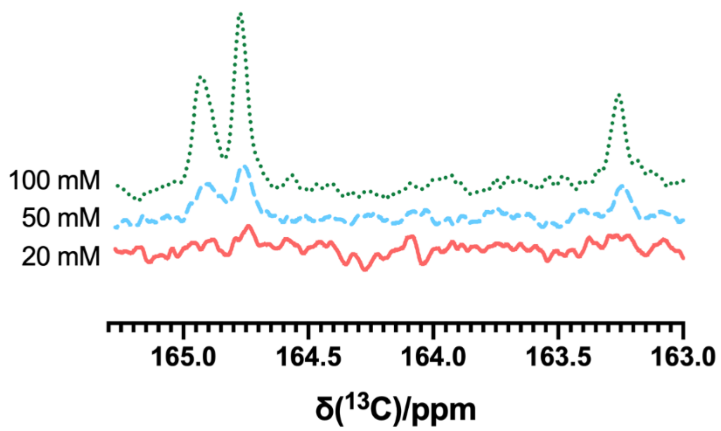

Fig. S1.

CO_2 forms carbamates on Ub. The carbamate signal intensity is affected by the concentration $NaH^{13}CO_3$. The carbamate region from 1D ^{13}C-NMR spectra is shown for buffers prepared with 1 mM Ub wild type varying $NaH^{13}CO_3$, and the intensities standardized to the Arg C_ζ resonances.

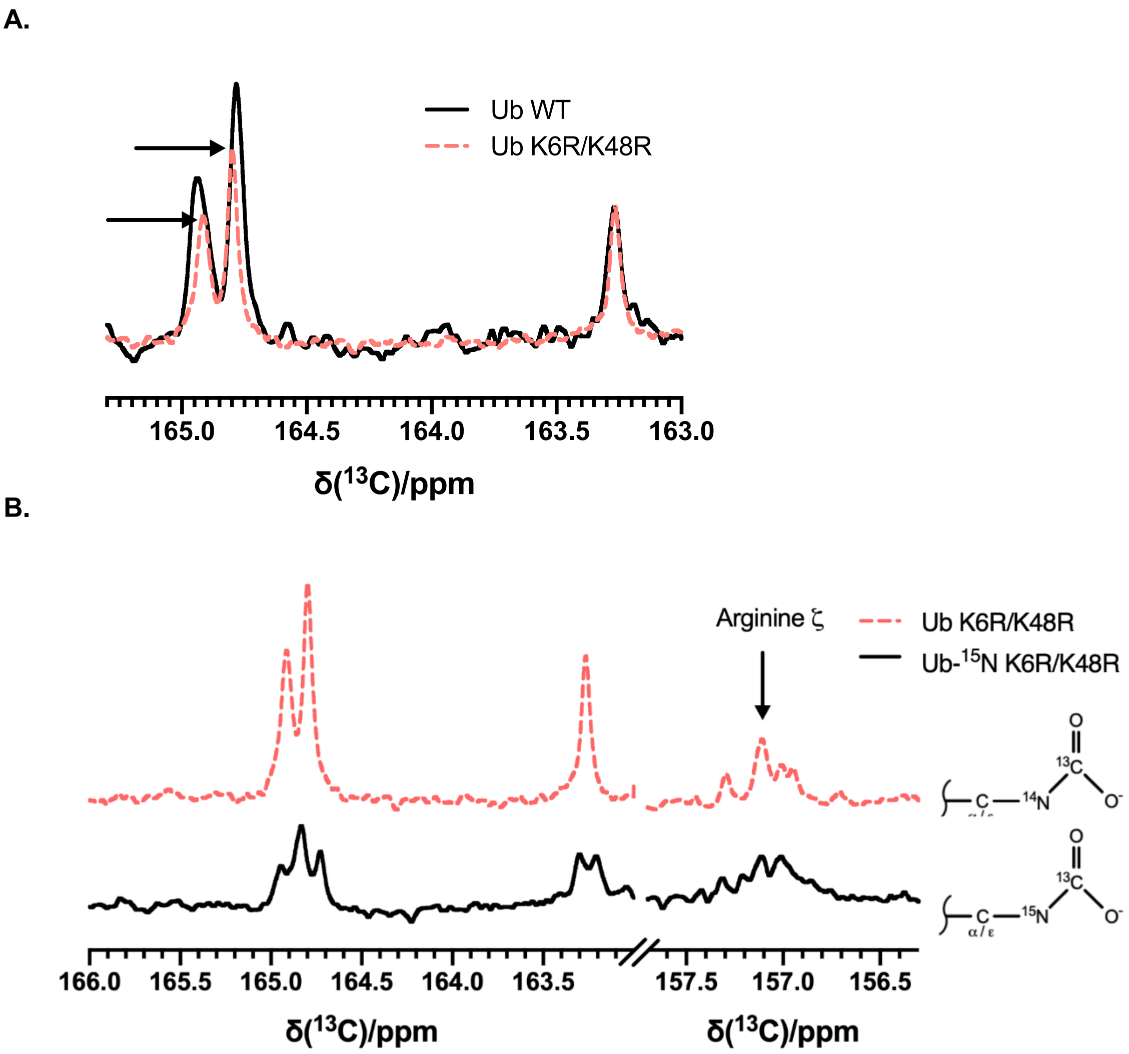

Fig. S2.

Ub exhibits five major carbamate resonances. **A**. The Ub K6R/K48R double mutant features a reduced intensity for two of the carbamate signals (indicated with arrows). **B**. The remaining carbamate resonances and those of arginine guanidinyl-carbons are split into doublets upon ^{15}N-Ub isotopic labeling; the splitting is consistent with a one-bond ^{13}C-^{15}N coupling of ~18.5 Hz.

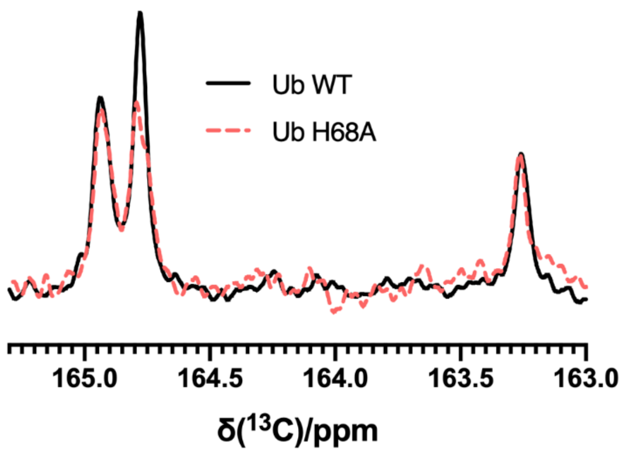

Fig. S3.
Ub exhibits five major carbamate resonances. A signal at 163.25 ppm was present in the Ub K0 mutant and was not ablated upon H68A mutation of the single Ub histidine.

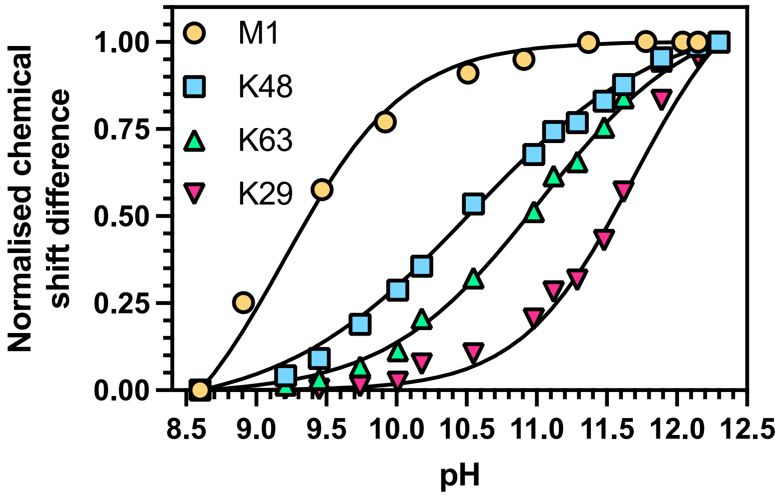

Fig. S4

Representative plots for the pH titrations of Ub amines. The data for K29, K48, and K63 side chain amines correspond to $^{15}N_{\zeta}$ chemical shifts recorded using the H2CN experiment, while the data for M1 correspond to $^{13}C_{\alpha}$ chemical shift recorded using the ^{1}H-^{13}C HSQC experiment. The lines represent the results of fitting the data to the Henderson-Hasselbalch model. The resulting pK_{aH} values are shown in Table 1. The chemical shifts were normalized using the following equation:

Normalized chemical shift difference = $(\delta_{low} - \delta_{obs}) / (\delta_{low} - \delta_{high})$,

where δ_{obs} is the observed chemical shift at a given pH value, while δ_{low} and δ_{high} are the chemical shifts of the same signal observed at the lower and upper bounds, respectively, of the pH range used in the titration.

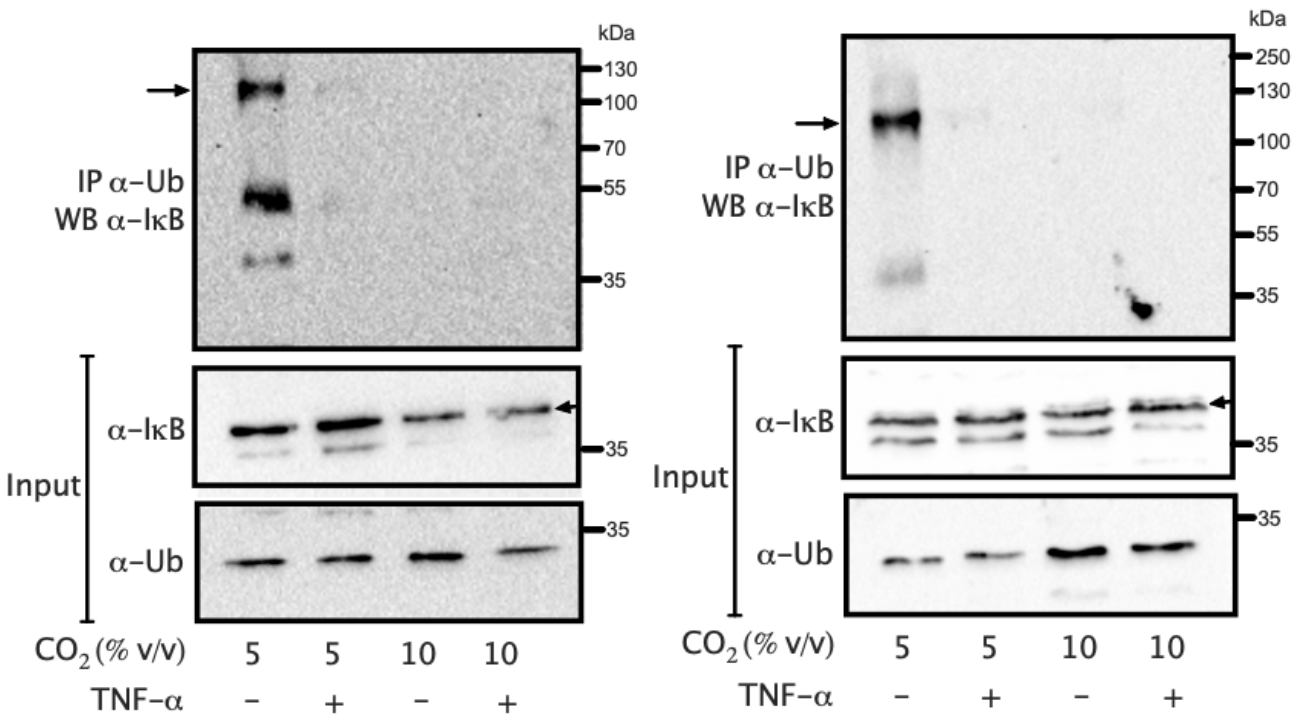

Fig. S5.
Interaction of IκB with Ub. Duplicate co-immunoprecipitation experiments of endogenous IκB and Ub from HEK 293 cells. The labels on the figure are as follows: input denotes an immunoblot of endogenous IκB and Ub; IP α-Ub WB α-IκB, an immunoblot performed using an α-IκB antibody after immunoprecipitation with an α-Ub antibody; CO_2 (% v/v) indicates gas conditions under which the experiment was performed; TNF-α indicates the presence or absence of 30 ng mL^{-1} TNF-α arrow head in α-IκB Input denotes IκB protein; arrow in top panel indicates IκB-Ub conjugate; kDa indicates molecular weight markers.

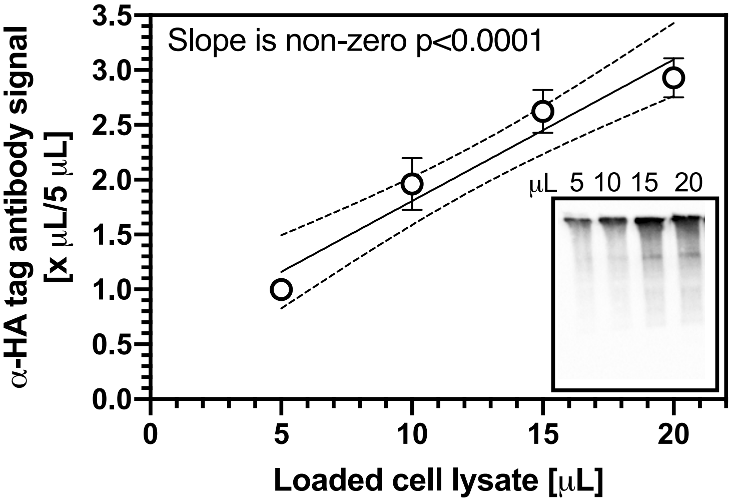

Fig. S6.
Plot of α-HA tag antibody signal against amount of loaded cell lysate. The α-HA tag antibody signal values of the y-axis are normalized to the signal with 5 μL of loaded cell lysate. The dotted line shows the 95% confidence interval for the linear regression. The slope of the linear regression is significantly non-zero and western blotting was performed with an amount of lysate with the boundaries of the plot. The inset shows an example immunoblot for the α-HA tag antibody signal with the indicated amount of loaded cell lysate.

Supplementary Excel File Captions

Figure 4B raw data (abi5507_Suppl._Excel_seq4_v1). Raw and processed fluorescent reporter counts for the plot of the ratio of fluorescence reporter activity for transfected NF-κB/293/GFP-LucTM cells at 5 and 10% (v/v) CO_2 treated with 30 ng mL-1 TNF-α.

Figure 4C raw data (abi5507_Suppl._Excel_seq5_v1). Raw and processed western blot band densities for the plot of the ratio of Ub expression at 5% (v/v) versus 10% (v/v) CO_2.

Figure 5B raw data (abi5507_Suppl._Excel_seq6_v1). Raw and processed p65 ELISA counts and western blot band densities.
